# DRMAAtic: dramatically improve your cluster potential

**DOI:** 10.1093/bioadv/vbaf112

**Published:** 2025-05-15

**Authors:** Alessio Del Conte, Hamidreza Ghafouri, Damiano Clementel, Ivan Mičetić, Damiano Piovesan, Silvio C E Tosatto, Alexander Miguel Monzon

**Affiliations:** Department of Biomedical Sciences, University of Padova, Padova 35131, Italy; Department of Biomedical Sciences, University of Padova, Padova 35131, Italy; Department of Biomedical Sciences, University of Padova, Padova 35131, Italy; Department of Biomedical Sciences, University of Padova, Padova 35131, Italy; Department of Biomedical Sciences, University of Padova, Padova 35131, Italy; Department of Biomedical Sciences, University of Padova, Padova 35131, Italy; Institute of Biomembranes, Bioenergetics and Molecular Biotechnologies, National Research Council (CNR-IBIOM), Bari 70126, Italy; Department of Biomedical Sciences, University of Padova, Padova 35131, Italy

## Abstract

**Motivation:**

The accessibility and usability of high-performance computing (HPC) resources remain significant challenges in bioinformatics, particularly for researchers lacking extensive technical expertise. While Distributed Resource Managers (DRMs) optimize resource utilization, the complexities of interfacing with these systems often hinder broader adoption. DRMAAtic addresses these challenges by integrating the Distributed Resource Management Application API (DRMAA) with a user-friendly RESTful interface, simplifying job management across diverse HPC environments. This framework empowers researchers to submit, monitor, and retrieve computational jobs securely and efficiently, without requiring deep knowledge of underlying cluster configurations.

**Results:**

We present DRMAAtic, a flexible and scalable tool that bridges the gap between web interfaces and HPC infrastructures. Built on the Django REST Framework, DRMAAtic supports seamless job submission and management via HTTP calls. Its modular architecture enables integration with any DRM supporting DRMAA APIs and offers robust features such as role-based access control, throttling mechanisms, and dependency management. Successful applications of DRMAAtic include the RING web server for protein structure analysis, the CAID Prediction Portal for disorder and binding predictions, and the Protein Ensemble Database deposition server. These deployments demonstrate DRMAAtic’s potential to enhance computational workflows, improve resource efficiency, and facilitate open science in life sciences.

**Availability and implementation:**

https://github.com/BioComputingUP/DRMAAtic, https://drmaatic.biocomputingup.it/.

## 1 Introduction

In contemporary scientific research, the dissemination of computational tools and algorithms through intuitive web interfaces has become essential for engaging a broad spectrum of users. In life sciences, particularly in bioinformatics and computational biology, web interfaces not only enhance accessibility but also facilitate reproducibility, enabling researchers worldwide to utilize advanced computational methods without needing extensive technical expertise or dedicated local infrastructure. The web server issue of the *Nucleic Acids Research* journal underscores the growing significance of web-based computational solutions for the biological community. For example, 89 articles were published in 2021 (Rigden and Fernández 2021), 102 in 2022 (Rigden and Fernández 2022), 81 in 2023 (Rigden and Fernández 2023), and 74 in 2024 (Rigden and Fernández 2024), highlighting the continued demand for streamlined job creation and execution in web-based environments. Moreover, the Galaxy Project (The Galaxy Community 2024) is an open-source, web-based platform designed to make computational biology accessible to researchers without programming expertise. It provides a user-friendly interface for constructing and executing complex bioinformatics workflows, emphasizing accessibility, reproducibility, and transparency in data analysis.

However, facilitating secure and efficient access for external applications to internal high-performance computing (HPC) clusters presents significant challenges. Traditional HTTP protocols are not inherently designed to manage the demands of long-running computational tasks that require substantial resources and often necessitate distribution across multiple nodes within a cluster. This limitation arises because HTTP operates on a request–response model, which is typically synchronous and does not accommodate prolonged operations well. For example, consider a scenario where a user submits a request to perform a resource-intensive data analysis or machine learning task. In such cases, implementing a solution often involves introducing mechanisms to handle the asynchronous nature of these tasks.

On the server side, the application must manage the job, spawning a new process, and monitor its progress and completion, all without waiting or blocking the main application process. This could involve checking if the results are available on the file system, querying a database or intra-process communication. These additional layers of logic introduce challenges related to state management, consistency, and fault tolerance, which traditional HTTP protocols do not natively address. This gap is compounded by existing web server solutions, which are typically tailored to specific use cases and lack the flexibility for general-purpose, customizable job execution.

Distributed Resource Managers (DRMs) are key in managing computational tasks across networked nodes. These systems optimize resource utilization through sophisticated scheduling and management policies, distributing tasks to maximize throughput and efficiency. The Distributed Resource Management Application API (DRMAA) (Troger *et al.* 2007) (https://drmaa.org/) complements these systems by offering a standardized interface for interacting with DRMs, abstracting their complexities to facilitate seamless job submission, control, and monitoring. Despite its potential, the adoption of DRMAA has been limited, with only a subset of DRMs fully implementing the latest standards. This disconnect hinders the development of interoperable tools that can bridge the gap between web-based interfaces and distributed computing environments.

Nowadays, the development of modern Web APIs is predominantly centered around the Representational State Transfer (REST) architecture (Fielding and Taylor 2000). A RESTful API is an implementation of an API that strictly adheres to the principles and constraints of REST architecture, following a standardized design pattern that defines a consistent and stateless set of operations. This architecture is particularly well-suited for web-based services, offering key advantages such as enhanced performance, scalability, and flexibility. RESTful Web APIs facilitate interoperability between systems and applications, making them a cornerstone of web software development (Cruz *et al.* 2020).

Several frameworks have been developed to expose HPC resources through RESTful APIs, notably NEWT (Cholia and Sun 2015), FirecREST (Cruz *et al.* 2020), and SCEAPI (Rongqiang *et al.* 2017). NEWT provides a web API for remote job submission, monitoring, and file system access on HPC systems. FirecREST takes a microservice-based approach to HPC middleware, handling job management and data transfer with tight integration into existing supercomputing infrastructure. SCEAPI offers a unified RESTful interface for job execution, file transfer, and authentication across multiple supercomputing centers. While these solutions significantly simplify HPC interactions, they are often tied to specific configurations and require users to understand cluster details, which can hinder accessibility for non-experts (see Table 1 for a more detailed comparison). In this work, we introduce DRMAAtic that fits into this ecosystem as a more flexible, user-centric alternative. It leverages the standardized DRMAA interface within a RESTful Django-based framework (DRF) to abstract away cluster-specific intricacies, enabling users to submit predefined computational tasks, monitor progress, and retrieve results via simple HTTP calls. By adopting a job-centric approach (with task templates and built-in result handling) and supporting robust authentication, DRMAAtic extends HPC capabilities to web applications and services with minimal user-side complexity, effectively bridging the gap between complex HPC environments and user-friendly web interfaces. Here, we present the architecture and functionalities of DRMAAtic, highlighting its potential as a key tool for organizations, research groups, and individual users seeking to enhance the accessibility and usability of computational resources.

**Table 1.** Comparative summary of DRMAAtic and other RESTful HPC API solutions.^a^

Framework	Ease of integration	Task control model	File handling	User management	Extensibility	Deployment complexity
**DRMAAtic**	(***)—standalone REST API (Django) easily integrates into web apps; minimal HPC knowledge needed for end-users.	Predefined tasks, simplifying submission but not permitting arbitrary jobs.	Built-in file staging and result retrieval via job context (outputs downloadable through API).	External auth support (e.g. OAuth/ORCID) with role-based access; users need not have local HPC accounts.	Designed for extension: new task types and parameters can be added; portable to any DRM via DRMAA.	(**)—runs as a single web service (Docker deployment available) on top of the cluster’s scheduler (requires DRMAA library).
**NEWT**	(**)—provides REST endpoints; integration requires users to have HPC accounts and knowledge of the target system.	General job submission (no preset tasks; users submit scripts or commands), offering flexibility with more user responsibility.	Direct file system access and transfer endpoints for upload/download and directory listing.	Tied to HPC center authentication (user accounts or API keys); uses existing user credentials for job execution.	Extensible via plugin framework to support new commands or resources.	(**)—a monolithic service that must be set up within the HPC environment; less complex than microservices but site-specific.
**FirecREST**	Variable-rich API for HPC operations, but primarily for portal developers; requires token-based access and HPC-center setup.	General job submission and system operations through microservices (no built-in task templates).	Dedicated microservices for data transfer (optimized for large files and HPC storage systems).	Integrates with HPC site identity management (tokens/OAuth with HPC accounts); enforces per-user permissions.	Loosely coupled microservices allow adding or upgrading components (e.g. new services) at the cost of greater system complexity.	(***)—complex microservice architecture (API gateway + multiple services); intended for deployment by large HPC centers.
**SCEAPI**	(**)—offers a unified API across sites; integration is easier for users once deployed, but setting up across multiple HPC centers is involved.	General job submission across federated resources (standard CRUD for jobs; no specific task templates).	File transfer included (endpoints for moving data to/from each site’s storage).	Centralized SSO and OAuth2 for multiple clusters; maps users to site accounts, ensuring security across centers.	Extensible to additional HPC centers and services within its federation model; not focused on user-defined task abstraction.	(***)—requires SCE middleware and coordination between HPC sites; suitable for national or multi-institutional HPC infrastructure.

a Asterisks indicate relative levels among frameworks as follows: (*) = Low, (**) = Moderate, and (***) = High.

## 2 Implementation

The core of the DRMAAtic architecture is the execution of a job in a DRM. Each job must be defined as an executable task with pre-configured parameters specifying how and where the task will be executed. These parameters also include a set of user-defined options provided at execution time. Users initiate job execution by selecting a task, providing necessary inputs, and submitting the job. The job is subsequently created and forwarded to the DRM for execution. A universally unique identifier (UUID) is generated for the job, allowing users to track its state and interact with it as needed.

### 2.1 Data modeling and management

**Figure 1.**
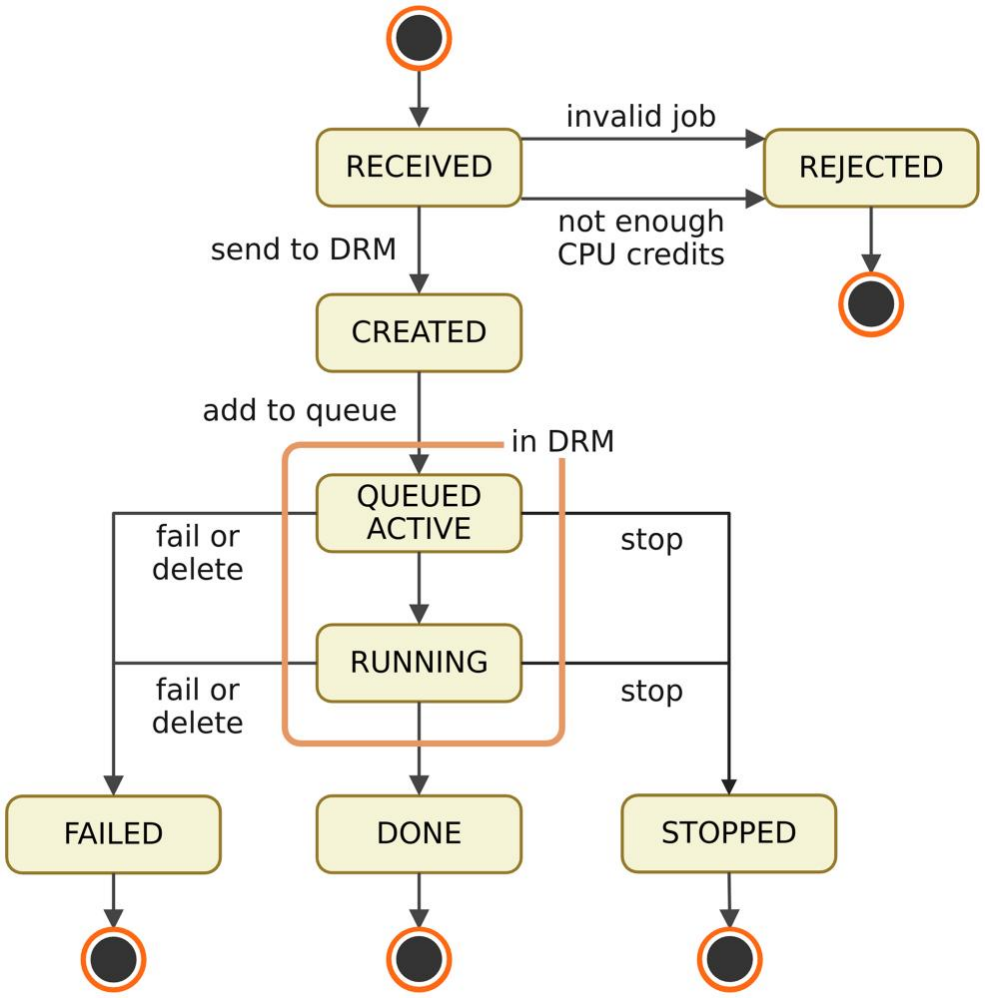
Execution flow of a job with its relative states. A job can have different states in its lifetime, depending on the user input or the result of its execution.

DRMAAtic leverages the Django REST Framework (DRF) and its Object-Relational Mapping (ORM) capabilities to model the underlying database structure. The database schema (Fig. 1, available as supplementary data at *Bioinformatics Advances* online) centers around the job entity, which includes critical attributes like a UUID, description, and creation timestamp. Jobs are interconnected with auxiliary entities, such as tasks to be executed, through relational mappings. Additionally, jobs may inherit execution directories by referencing parent jobs, and the “job_dependencies” table enables the specification of complex inter-job relationships essential for constructing dependency-driven workflows.

Tasks are defined by attributes such as a unique identifier, execution command, queue assignment, CPU, and memory requirements. Each task can include parameters with a variety of types (e.g. Boolean, integer, float, string, or file) and features such as default values, administrator-only visibility, and required status. User groups further refine execution permissions, facilitating resource allocation via throttling rates and execution tokens.

### 2.2 API endpoints and user interaction

**Figure 2.**
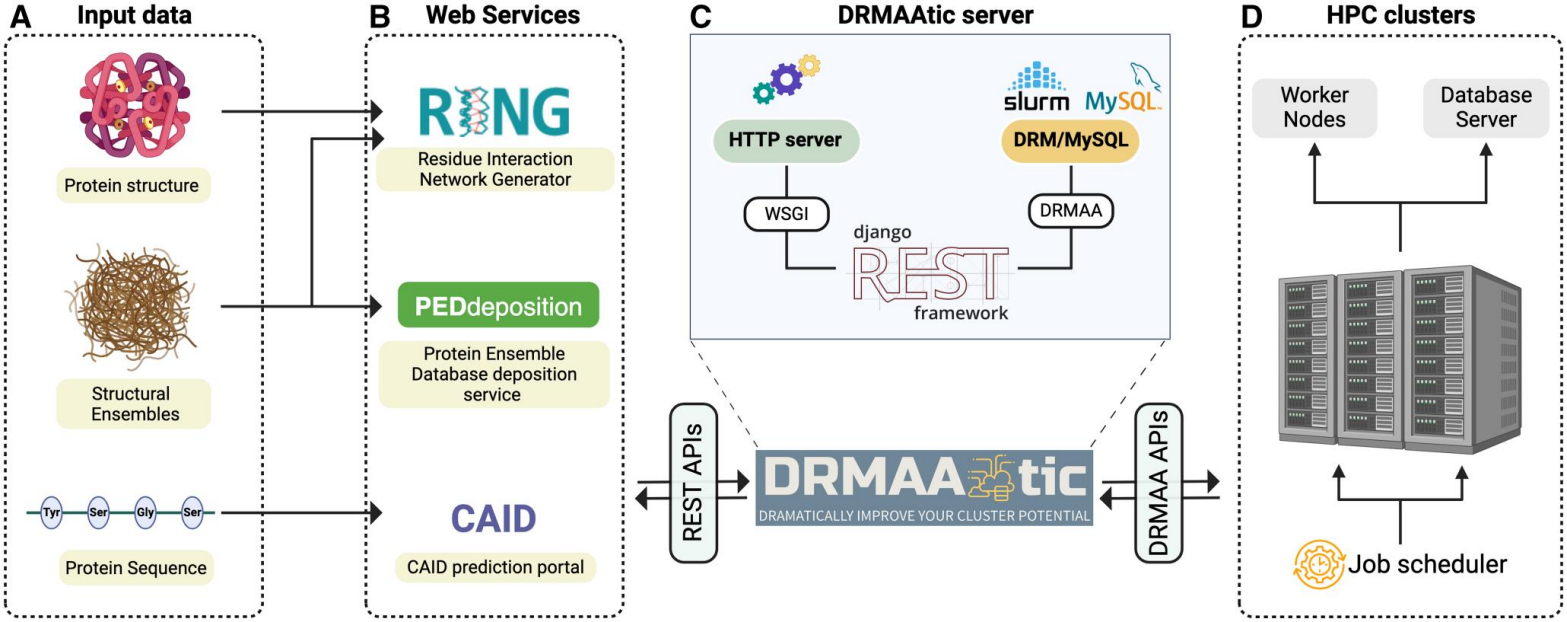
DRMAAtic workflow and architecture. (A) Input data from user-facing web interfaces. Examples of web services utilizing these inputs are RING, which accepts protein structures and conformational ensembles, PEDdeposition for structural ensemble submission, and CAID for sequence-based predictions. (B) Web services act as clients making REST API calls to DRMAAtic (from front-end, not back-end), facilitating input submission and delivering results. (C) DRMAAtic translates requests into job submissions to the HPC cluster, leveraging the functionality of DRMAA with RESTful APIs, implemented through the Django REST Framework. (D) Job execution and tracking occur on worker nodes, with metadata stored in the backend database.

API endpoints in DRMAAtic are implemented using DRF views, enabling easy interactions through HTTP requests. These endpoints cover the full spectrum of job lifecycle management, including submission, monitoring, and retrieval of results (Table 2). For instance, a user submits a job by issuing a POST request to the /*job* endpoint. This triggers server-side validation of input parameters, job creation, and DRM submission, with a structured response containing job metadata. Users may also leverage endpoints for querying job states, downloading outputs, and managing dependencies. An example (Fig. 2, available as supplementary data at *Bioinformatics Advances* online): a user submitting a job can specify a list of parameters in the request body, along with the task name to be executed and optional fields such as parent_job, dependencies, and dependency_type.

**Table 2.** DRMAAtic API endpoints.^a^

Method	Route	Description
GET	/{provider}/token/	Allows users to obtain JWT tokens for authenticating with DRMAAtic, using access tokens from external providers.
GET	/cpu-credit/	Provides the amount of available CPU credits for the user.
POST	/job/	Enables users to create new jobs, defining job parameters based on specific tasks.
GET	/job/	Allows users to list and query jobs, including filtering by state, job name, and other criteria.
GET	/job/{uuid}/	Retrieves all the metadata of the job using its UUID, updating its state if it is not in a final state.
DELETE	/job/{uuid}/	Permits users to delete specific jobs, stopping them in DRM while preserving the execution directory and the metadata.
GET	/job/{uuid}/download/	Allows users to download a zip file containing all the output files of a job.
GET	/job/{uuid}/file/	Lists all the output files of a job, including their paths.
GET	/job/{uuid}/file/{path}	Enables users to download a specific output file of a job, given its path in the output directory.
GET	/job/{uuid}/status/	Provides the state of a job, updating it if necessary.
PUT	/job/{uuid}/stop/	Stops a job that is executing in the DRM, returns an error if the job was already executed.
GET	/task/	Lists all available tasks in the DRMAAtic system, including their parameters and descriptions.
GET	/task/{name}/	Retrieve a specific task.

a The first column specifies the HTTP method allowed for the endpoint. The second column, the route, provides the address of the endpoint where the client should send the request (the root indicates the targeted resource). Lastly, the description column explains the purpose and functionality of the endpoint.

Detailed API documentation, enriched with Swagger-based descriptions, is available at URL: https://drmaatic.biocomputingup.it to facilitate user adoption.

### 2.3 Authentication and authorization

DRMAAtic employs external authentication services, such as ORCID (URL: https://orcid.org/), for identity verification, while handling resource authorization internally. Authentication is facilitated via OAuth 2.0 implicit flow, where access tokens are converted into stateless JSON Web Tokens (JWTs). Upon receipt of a valid JWT in an HTTP header, DRMAAtic authenticates the user, retrieves metadata from the authentication provider, and ensures secure interaction (Fig. 3, available as supplementary data at *Bioinformatics Advances* online).

Sensitive resources and endpoints are protected using role-based access control, ensuring that only authorized users can access specific functionalities. This dual-layered approach enhances security while maintaining a robust user experience.

### 2.4 Throttling and resource management

DRMAAtic employs a dual-throttling strategy to ensure efficient and fair use of HPC resources. API request throttling limits the rate of user interactions to prevent unnecessary strain on the system. More critically, job submission is governed by a CPU token-based mechanism. Each user is assigned a fixed-size CPU token bucket (e.g. 100 tokens), which is replenished at a defined rate (e.g. 10 tokens per hour). Jobs can be configured to consume tokens on submission proportional to their CPU needs, for example, a task requiring 4 CPUs will consume 4 tokens. If a user lacks sufficient tokens, the job submission is deferred with a message indicating the wait time until enough tokens are available (Fig. 4, available as supplementary data at *Bioinformatics Advances* online). The system identifies users either through the authentication mechanism or, if they are not authenticated, by their IP address.

In high-demand scenarios, this mechanism effectively smooths submission spikes, preventing overloads on the DRM. The throttling system guarantees that users cannot monopolize cluster resources, maintaining responsiveness for all users. Internal tests showed that even under concurrent multi-user submissions (e.g. 50+ jobs across 5 users), DRMAAtic maintained consistent job handling without system crashes or degraded service. When the cluster reaches full load, new job requests are queued by the DRM, while DRMAAtic provides informative status updates without blocking the interface.

### 2.5 Administration and configuration interface

DRMAAtic provides an administration web interface accessible via a dedicated “/admin” route (Fig. 5, available as supplementary data at *Bioinformatics Advances* online). Administrators can configure execution tasks, queues, and user groups using this interface. Through the web-based administration interface, an administrator can define new tasks by specifying their name, execution command, associated computational requirements (e.g. memory, CPUs, queue), and any input parameters required at runtime. Parameters can be customized with type validation (e.g. integer, float, file) and can include optional flags to modify the execution behavior. The interface also enables monitoring of submitted jobs, parameter configurations, and debugging processes (Fig. 6, available as supplementary data at *Bioinformatics Advances* online).

Additionally, administrators can manage system performance and security by configuring throttling parameters, execution tokens, and the longevity of access tokens. This low-code configuration process enables seamless integration of existing scripts or tools with minimal technical overhead, making DRMAAtic accessible even for non-expert system administrators.

## 3 Results

In this section, the general job execution workflow is described, along with the integration of DRMAAtic with the SLURM workload manager. Finally, three application examples utilizing DRMAAtic as a core component for job execution are presented and discussed.

### 3.1 Job execution workflow

The job lifecycle in DRMAAtic encompasses multiple states, ensuring systematic management from submission to completion (Fig. 1). Each state is uniquely defined and linked to its management context, either by the web server or the underlying DRM. Upon job receipt, the server validates input parameters to prevent computational errors and resource misuse. Jobs failing to meet predefined constraints, such as incorrect configurations or insufficient CPU credits, are rejected to protect cluster resources and maintain availability.

Validated jobs are forwarded to the DRM, where the scheduler assigns them to the cluster queue and marks them as “queued active.” Jobs transition to the “running” state when execution begins. On successful completion (exit code zero), jobs are marked as “done.” Errors or disruptions during execution result in a “failed” state, while users can issue stop commands to halt active jobs, transitioning them to the “stopped” state.

DRMAAtic dynamically tracks job states through direct queries to the DRM during user requests, avoiding resource-intensive polling. The server updates job states in its database and communicates them to users in real time. Final states (done, failed, stopped, rejected) are preserved in the DRMAAtic database without further DRM updates, minimizing unnecessary system interactions. This structured workflow ensures efficient job management and optimal resource utilization while maintaining efficient user interaction with the DRM.

### 3.2 DRMAAtic and SLURM integration

As previously highlighted, DRMAAtic is designed to integrate smoothly with any DRM system that supports or exposes DRMAA APIs (Fig. 2C and D). For this study, our focus was on integrating DRMAAtic with SLURM, a widely adopted workload manager in HPC environments. DRMAAtic’s Python-based implementation utilizes the DRMAA-Python library (available at https://github.com/pygridtools/drmaa-python) to access DRMAA bindings directly, enabling efficient job submission and management.

To ensure cross-compatibility, we developed a job structure that abstracts job configurations from DRM-specific implementations. This abstraction allows DRMAAtic to incorporate additional SLURM-specific parameters, referred to as “native specifications,” without deviating from the DRMAA v1 standard. Key native specifications included in DRMAAtic are CPU-per-task allocation, minimum RAM requirements, and partition or queue designations. These enhancements ensure robust job configuration while maintaining the flexibility to integrate other DRMs in the future. Notably, the nature and extent of native specifications vary across DRMs, determined by their respective implementations of DRMAA bindings.

#### 3.2.1 Deploying DRMAAtic with Docker

Deploying DRMAAtic in a production environment requires proper configuration of the cluster and its dependencies. For simplified local testing, a Docker Compose configuration was developed to provide all necessary services:

- MariaDB: The primary relational database management system utilized by both SLURM and DRMAAtic.
- phpMyAdmin: A web-based interface that simplifies MariaDB administration.
- slurmdbd: SLURM’s database daemon, responsible for job accounting and tracking.
- slurmctld: The central management component in SLURM, overseeing job scheduling, resource allocation, and cluster monitoring.
- Worker Nodes (c1 and c2): Nodes configured to execute computational tasks within the cluster.
- DRMAAtic: Serves as the interface to SLURM via the DRMAA API, using MariaDB for data storage.

During the initial execution of the Docker Compose setup, a preconfigured test database is deployed, which includes configurations for a sample task and an administrator account. This enables immediate functionality testing of DRMAAtic. For production environments, DRMAAtic can be deployed as a Docker container interfacing with an existing SLURM control daemon. In such cases, careful configuration of SLURM is essential to ensure robust communication with the control machine. Additionally, deploying DRMAAtic in production requires a HTTP server (e.g. Apache) paired with a Web Server Gateway Interface (WSGI) (Fig. 2C). This setup provides the stability, scalability, and security required for production, including features such as SSL/TLS termination and advanced access control.

File and Directory Management: Docker-based deployments of DRMAAtic require proper handling of job-specific files and directories within mounted volumes. Ensuring that the user executing jobs has appropriate write permissions is critical. The internal user in the Docker container must correspond to an external user to prevent permission conflicts, particularly when Docker user remapping is enabled. Addressing this challenge involves creating a dedicated user whose UID is synchronized with external users across all worker nodes.

### 3.3 Application examples

DRMAAtic supports client-side mashup integration, where REST API calls originate from the web interface front-end (e.g. JavaScript or React apps) rather than backend middleware. This architecture enables seamless integration into web portals, aligning with modern Web 2.0 principles.

#### 3.3.1 RING web-server

The first operative web application interacting with the DRMAAtic web server is the Residue Interaction Network Generator (RING) (URL: https://ring.biocomputingup.it/). RING web server allows users to execute the RING software (Del Conte *et al.* 2024) remotely on a cluster, eliminating the need for local installation. Moreover, the server presents results interactively, enabling users to analyze Residue Interaction Networks (RINs), representing the interactions between residues within a protein structure.

The UI allows users to upload a protein structure or structural ensemble for analysis or retrieve it from Protein Data Bank (Berman *et al.* 2003) and AlphaFoldDB (Varadi *et al.* 2024). Additionally, users can define various parameters for the RING software, customizing the analysis and setting thresholds for identifying interactions. Upon job submission, the user-defined parameters are packaged and sent to DRMAAtic for validation. Once validated in the back-end, a RING task is executed within the DRM using the specified inputs and parameters. The server then identifies the job with a UUID, enabling users to monitor its status and retrieve output files upon completion.

Upon successful execution, the outputs are downloaded and processed for integration into interactive components of the UI such as graphs, 3D protein structure representations, and contact heatmaps. DRMAAtic’s architecture ensures efficient user and job management, cluster execution of RING tasks, and easy retrieval of prior jobs and their outputs.

#### 3.3.2 CAID Prediction Portal

The CAID Prediction Portal (Del Conte *et al.* 2023; https://caid.idpcentral.org/portal) exemplifies the advanced use of DRMAAtic for bioinformatics workflows. This portal accepts a protein sequence, job description, and optional email address as inputs, running disorder and binding prediction algorithms to identify disordered regions and binding sites. The portal integrates 40 distinct predictors from the literature, each executable against the query sequence. To enhance user experience, completed predictions are made available for download or visualization on the web page, managing different execution times across predictors.

The implementation strategy leverages DRMAAtic to manage dependencies systematically. A “main job” validates and organizes input data within the execution directory, spawning individual prediction tasks as dependent jobs. This approach ensures predictions are executed sequentially, with dependencies handled via DRMAAtic’s “afterok” mechanism for predictors requiring supplementary inputs. Each predictor generates outputs in its designated directory, populating the main job’s execution directory with comprehensive results.

The frontend employs UUIDs to track jobs, enabling an easy retrieval of related tasks. A polling mechanism monitors job states, automatically downloading results upon completion. If an email address is provided, a notification job is triggered to alert the user when all tasks have concluded, even in cases of partial failure, utilizing DRMAAtic’s “afterany” dependency setting.

This systematic and modular approach underscores the flexibility of DRMAAtic, ensuring efficient resource utilization and user-centric results presentation for complex bioinformatics tasks.

#### 3.3.3 Deposition web-server of Protein Ensemble Database

The Protein Ensemble Database (PED) (Ghafouri *et al.* 2024) serves as the main repository for structural ensembles of intrinsically disordered proteins (IDPs). Ensemble deposition is facilitated through a dedicated web server (URL: https://deposition.proteinensemble.org/), allowing users to submit structural ensemble data and execute the ensemble validation pipeline.

The user interface (UI) supports input of ensemble metadata, uploading structural ensemble files, and, when available, the weights of individual conformations. The backend efficiently manages this data and integrates with DRMAAtic via its API, enabling automated job submissions for ensemble validation and statistical analysis. Tools such as MolProbity (Williams *et al.* 2018), DSSP (Kabsch and Sander 1983), and relative solvent accessibility (RSA) calculations, among other global parameters derived from structural data, are used to process and evaluate the ensembles.

The backend facilitates interaction with DRMAAtic and updates the deposition database with job statuses in real time. Upon completion, result files are securely stored in protected storage, ensuring long-term preservation even after the associated DRMAAtic jobs are deleted. The integration between the backend and DRMAAtic improves the deposition process, keeping calculations and data processing nearly invisible to users until results are finalized and made available through the UI.

## 4 Discussion

The expanding role of web applications in life sciences, particularly in bioinformatics, highlights the importance of tools that simplify access to complex computational infrastructures. One of the main challenges in this domain is the heterogeneity of cluster architectures and resource managers, which makes web-based integration with HPC systems harder.

DRMAAtic addresses this challenge through its job-centric design, which emphasizes predefined task templates, simplified job submission, secure authentication, and intuitive result retrieval. Built on the DRF, DRMAAtic offers a modular, scalable platform with native support for the DRMAA standard, enabling compatibility with multiple DRM such as SLURM and PBS. Its architecture supports external authentication (e.g. ORCID) and role-based access control, making it suitable for public-facing services that do not rely on local Unix user accounts. Throttling and token-based resource allocation mechanisms further ensure fair usage in multi-user environments.

When compared to existing HPC REST APIs such as NEWT, FirecREST, and SCEAPI, DRMAAtic offers distinct advantages and trade-offs. It is ideal for web portals or science gateways where users interact with predefined workflows, requiring minimal technical knowledge. In contrast, NEWT and FirecREST support arbitrary job submissions and broader system access but are more complex to use and deploy, particularly for non-experts. DRMAAtic also simplifies integration into web mashups, thanks to its lightweight, self-contained architecture. While FirecREST employs a microservice model best suited for large HPC centers, and SCEAPI supports cross-institutional federation, DRMAAtic caters to individual labs or institutes needing a focused and easy-to-deploy solution.

From a security and usability standpoint, DRMAAtic allows users to submit and monitor jobs without requiring local accounts, while still maintaining control over access policies. File management is tightly linked to each job, allowing users to retrieve results without needing to navigate complex storage systems. While this limits flexibility in some advanced data workflows, it greatly enhances usability for predefined pipelines.

The versatility of DRMAAtic is demonstrated through its integration into real-world applications such as the RING web server, the CAID Prediction Portal, and the PED deposition system. These deployments showcase its ability to manage complex and interdependent workflows efficiently, all while providing an accessible user experience. DRMAAtic’s integration with SLURM, combined with its Docker-based deployment option, further emphasizes its adaptability and ease of maintenance.

In summary, DRMAAtic is most suitable when ease of integration, controlled job execution, and broad accessibility are top priorities, for instance, in biomedical or life sciences research portals that require HPC-backed computation through user-friendly web interfaces. It may be less appropriate in scenarios demanding unrestricted HPC access, deep system integration, or federation across HPC centers, where more comprehensive solutions like FirecREST, NEWT, or SCEAPI may be more aligned with user needs. DRMAAtic’s capacity to foster collaboration, improve accessibility, and optimize resource efficiency aligns with the overarching goals of open science and reproducibility in life sciences. As the demand for user-friendly and versatile web-based tools continues to grow, DRMAAtic represents a significant step toward addressing these evolving needs.

## Supplementary Material

vbaf112_Supplementary_Data

## Data Availability

The data underlying this article are available in *GitHub* at https://github.com/BioComputingUP/DRMAAtic.
