# Supplementary material for: DRMAAtic: dramatically improve your cluster potential
Source: Bioinform Adv. 2025 May 15;5(1):vbaf112. doi: 10.1093/bioadv/vbaf112 (PMC12133261; doi:10.1093/bioadv/vbaf112)
Supplement: vbaf112_Supplementary_Data [file vbaf112_supplementary_data.docx]

Supplementary Material

**DRMAAtic: dramatically improve your cluster potential**

Alessio Del Conte^1^, Hamidreza Ghafouri^1^, Damiano Clementel^1^, Ivan Mičetić^1^, Damiano Piovesan^1^, Silvio C.E. Tosatto^1,2,*^, Alexander Miguel Monzon^1,*^

^1^Department of Biomedical Sciences, University of Padova, Padova 35121, Italy

^2^Institute of Biomembranes, Bioenergetics and Molecular Biotechnologies, National Research Council (CNR-IBIOM), Bari 70126, Italy

*Corresponding authors. Department of Biomedical Sciences, University of Padova, Padova 35121, Italy. E-mails: alexander.monzon@unipd.it (A.M.M.) and silvio.tosatto@unipd.it (S.C.E.T.)


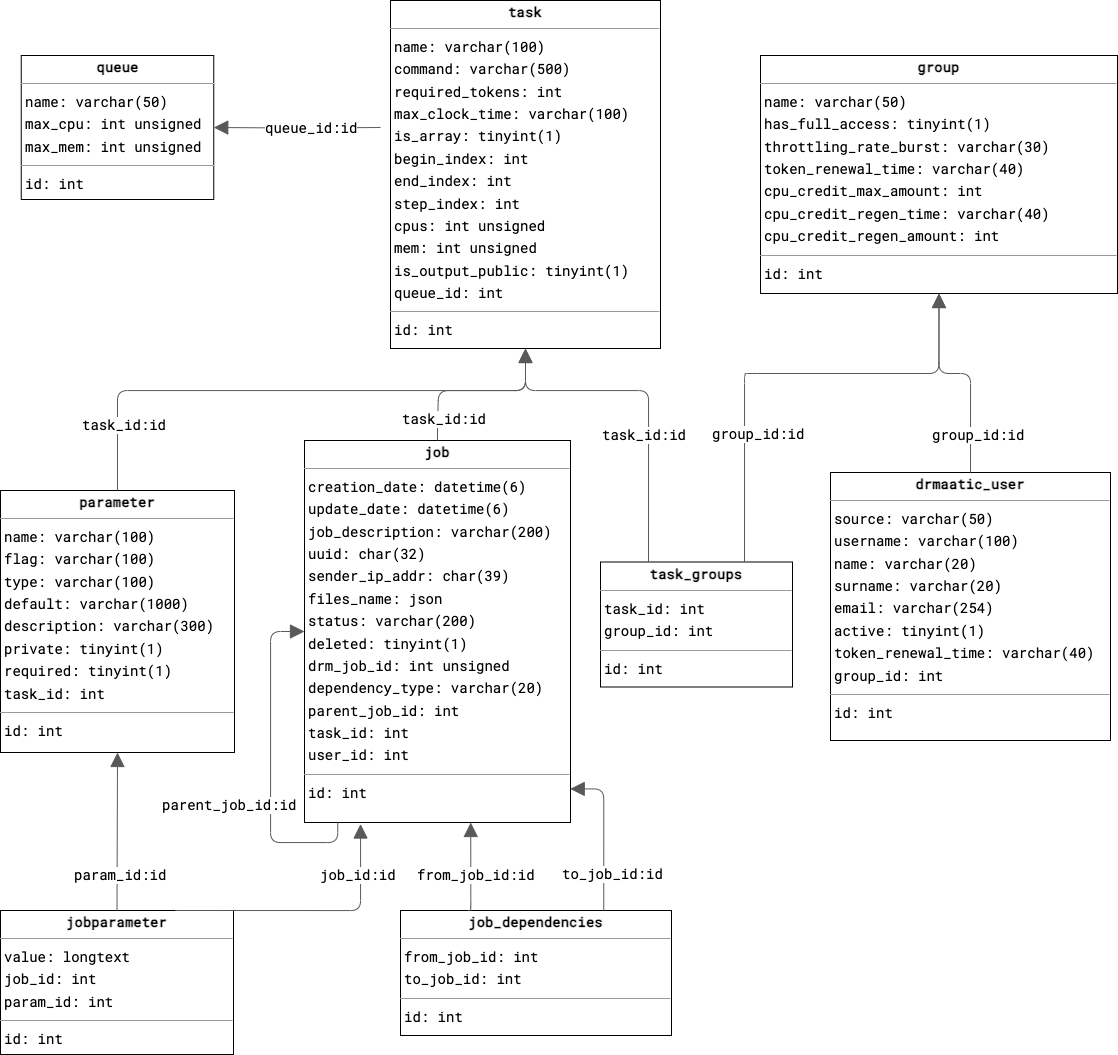


**Figure 1.** Database schema of DRMAAtic with all the models represented as rectangles, with a list of their fields. Arrows indicate the relation between two models, the label on the arrow is the foreign key that is used for the relation.

**
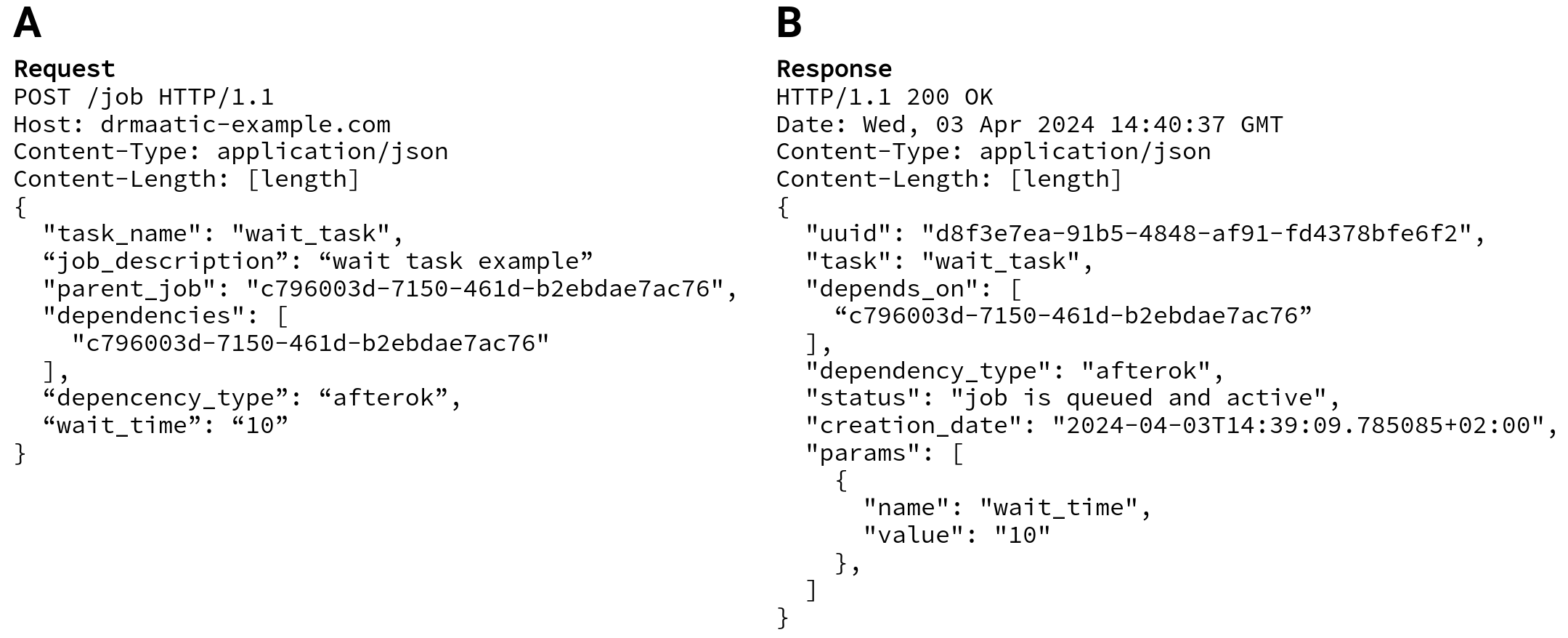
**

**Figure 2.** Example of job submission with a POST method, where the user wants to execute the *wait_task* that has a *parent_job* which also depends on, meaning that it will be executed in the same execution directory of the parent and only if the parent job will complete without any errors. The *wait_time* is a parameter for this specific task, that will be passed and used by the task script. In panel (A) is the request made from the client, in panel (B) the response with the information about the job.


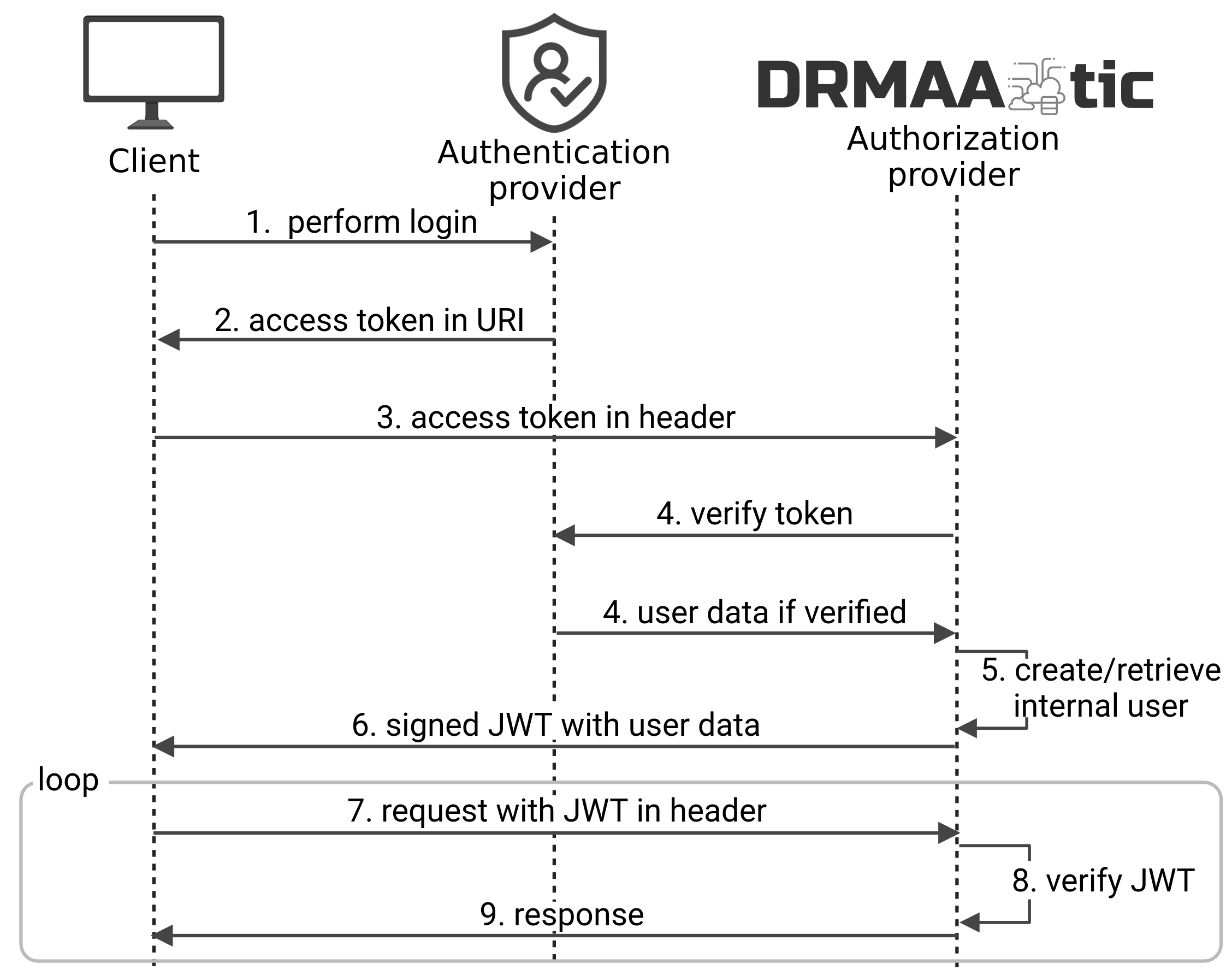


**Figure 3.** Scheme of the Oauth 2.0 implicit flow integrated in DRMAAtic with a JWT as authorization token. The authentication provider in this first implementation is the ORCID platform. URI: Uniform resource identifier.


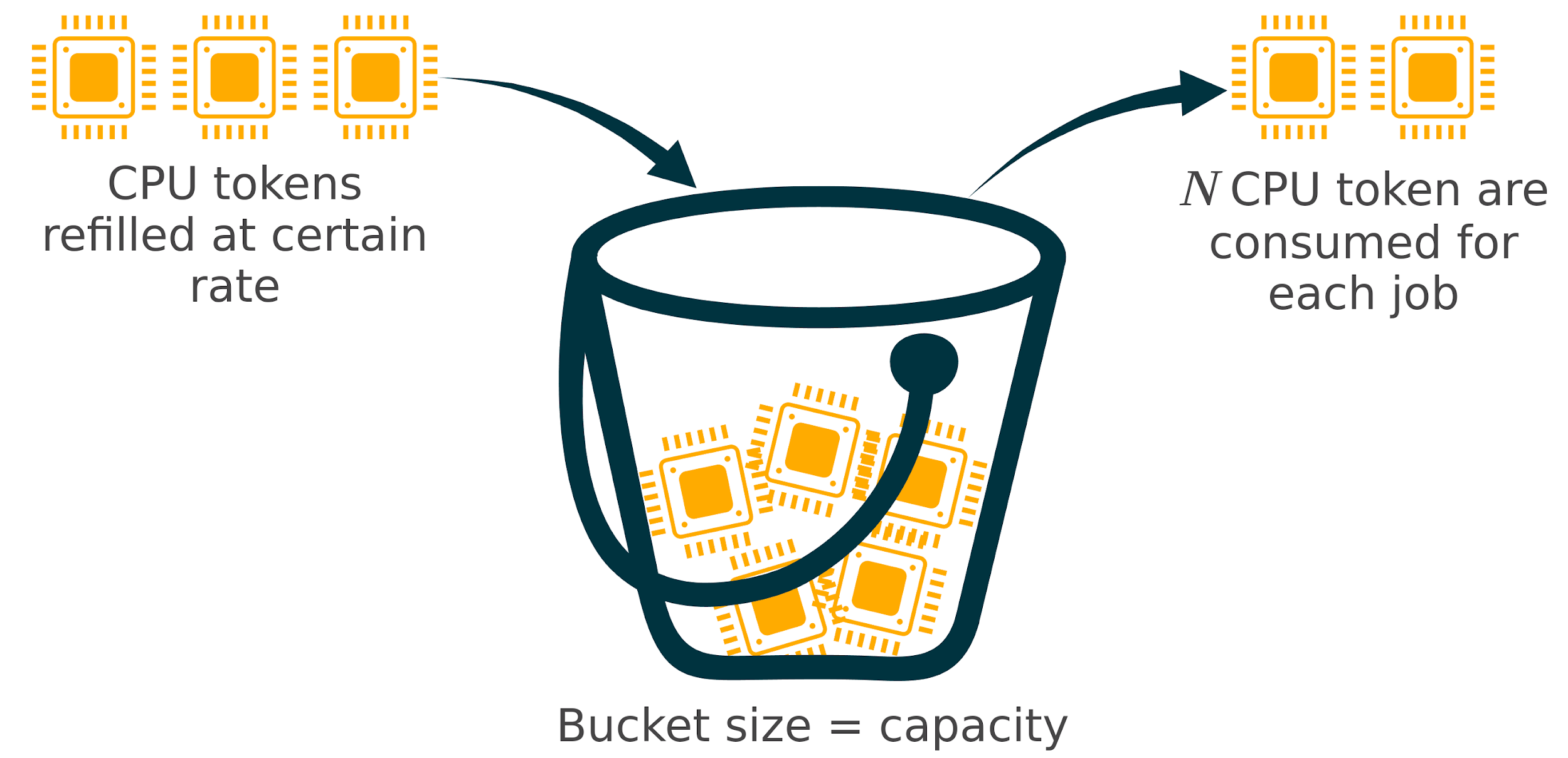


**Figure 4.** Scheme of how the CPU token bucket throttling algorithm works. To each user, identified by their IP address or authentication, a fixed number of CPU tokens is assigned. To each task there is a number of required tokens that need to be consumed upon job submission. If there are not enough tokens, the request is throttled. The bucket has a maximum size that gets replenished at a fixed rate.


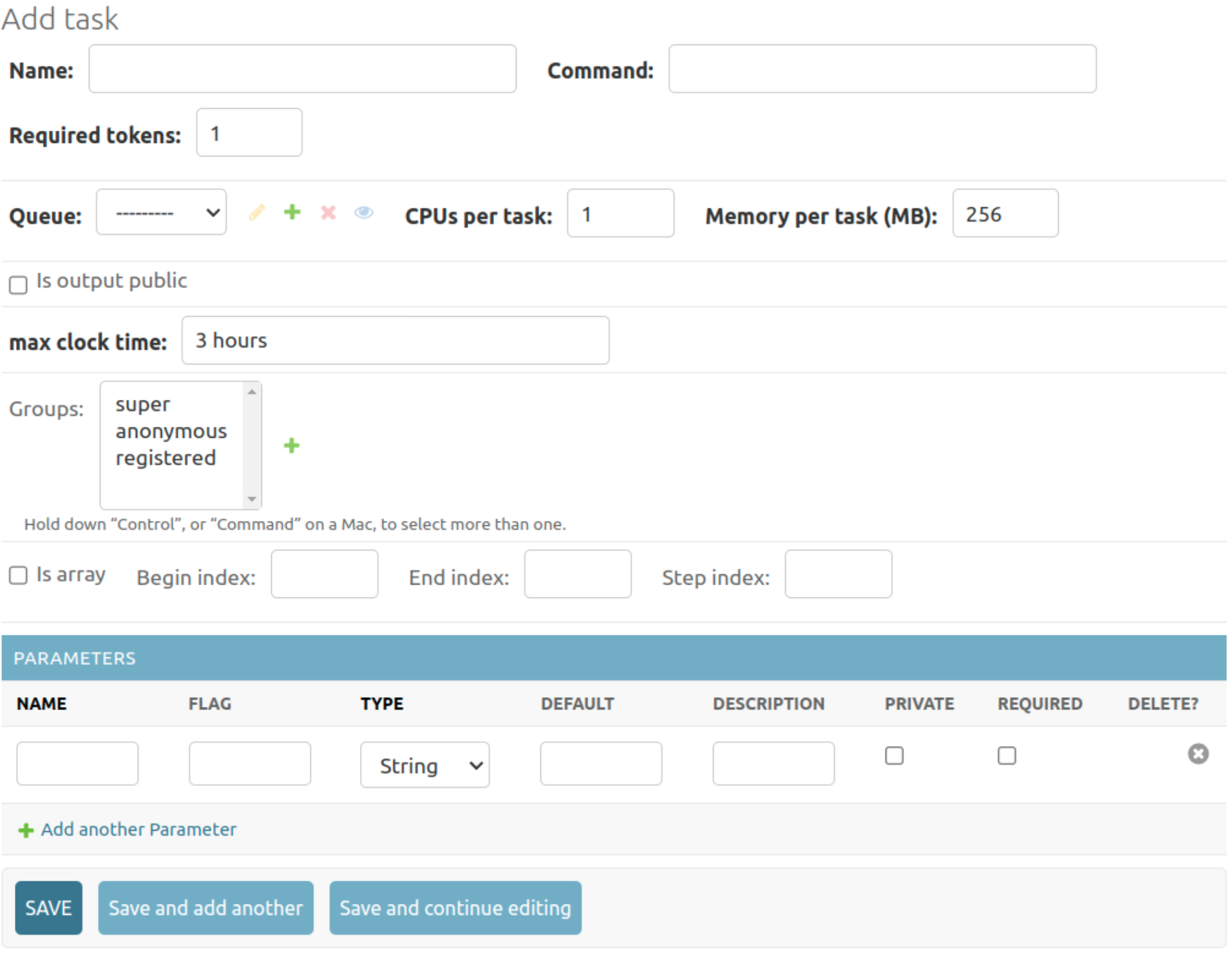


**Figure 5.** DRMAAtic Administration Interface: Adding a New Task. The administrator is prompted to complete the necessary fields (highlighted in bold). Subsequently, a parameter list can be configured to be passed the command, requiring each parameter to have a designated name and type. Additionally, if a flag is specified, it will be placed before the parameter value.


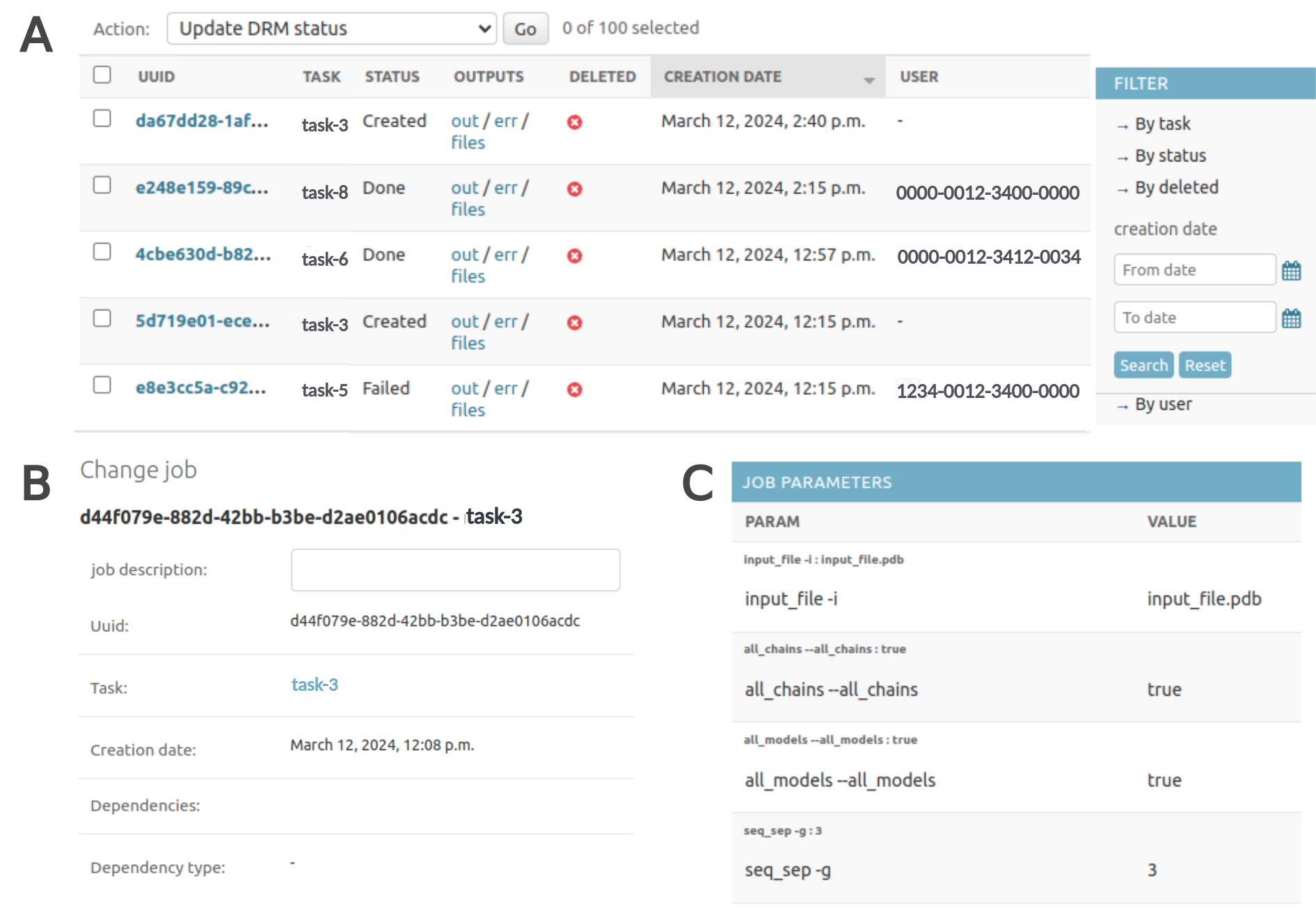


**Figure 6.** Interface for Job Administration: (A) presents a comprehensive list of submitted jobs, displaying task execution status, current state, outputs, and relevant details, with available filter options. (B) offers detailed job information, while (C) highlights input parameter values.
